# Initial study on quantitative electroencephalographic analysis of bioelectrical activity of the brain of children with fetal alcohol spectrum disorders (FASD) without epilepsy

**DOI:** 10.1038/s41598-022-26590-4

**Published:** 2023-01-03

**Authors:** Waldemar Bauer, Katarzyna Anna Dylag, Adam Lysiak, Wiktoria Wieczorek-Stawinska, Mariusz Pelc, Miroslaw Szmajda, Radek Martinek, Jaroslaw Zygarlicki, Bożena Bańdo, Monika Stomal-Slowinska, Aleksandra Kawala-Sterniuk

**Affiliations:** 1 Department of Automatic Control and Robotics, AGH University of Science and Technology, 30-059 Kraków, Poland; 2 St. Louis Children Hospital in Krakow, 30-663 Kraków, Poland; 3 Department of Pathophysiology, Jagiellonian University in Krakow – Collegium Medicum, 31-121 Kraków, Poland; 4 Faculty of Electrical Engineering, Automatic Control and Informatics, Opole University of Technology, 45-758 Opole, Poland; 5 School of Computing and Mathematical Sciences, University of Greenwich, London, SE10 9LS UK; 6 Department of Cybernetics and Biomedical Engineering, VSB—Technical University Ostrava—FEECS, 708 00 Ostrava-Poruba, Czech Republic; 7 Department of Neurosurgery, Vital Medic, 46-200 Kluczbork, Poland

**Keywords:** Computational biology and bioinformatics, Computational neuroscience, Data processing, Statistical methods

## Abstract

Fetal alcohol spectrum disorders (FASD) are spectrum of neurodevelopmental conditions associated with prenatal alcohol exposure. The FASD manifests mostly with facial dysmorphism, prenatal and postnatal growth retardation, and selected birth defects (including central nervous system defects). Unrecognized and untreated FASD leads to severe disability in adulthood. The diagnosis of FASD is based on clinical criteria and neither biomarkers nor imaging tests can be used in order to confirm the diagnosis. The quantitative electroencephalography (QEEG) is a type of EEG analysis, which involves the use of mathematical algorithms, and which has brought new possibilities of EEG signal evaluation, among the other things—the analysis of a specific frequency band. The main objective of this study was to identify characteristic patterns in QEEG among individuals affected with FASD. This study was of a pilot prospective study character with experimental group consisting of patients with newly diagnosed FASD and of the control group consisting of children with gastroenterological issues. The EEG recordings of both groups were obtained, than analyzed using a commercial QEEG module. As a results we were able to establish the dominance of the alpha rhythm over the beta rhythm in FASD-participants compared to those from the control group, mostly in frontal and temporal regions. Second important finding is an increased theta/beta ratio among patients with FASD. These findings are consistent with the current knowledge on the pathological processes resulting from the prenatal alcohol exposure. The obtained results and conclusions were promising, however, further research is necessary (and planned) in order to validate the use of QEEG tools in FASD diagnostics.

## Introduction

The term “fetal alcohol spectrum disorders” (FASD) encompasses a spectrum of neurodevelopmental conditions associated with prenatal alcohol exposure (PAE)^1,2^ manifestations of FASD are facial dysmorphism^3,4^, prenatal and postnatal growth retardation^5–7^ and selected birth defects^8–11^. It is the central nervous system, affected to the greatest extent, which manifests in different ways—from major structural defects^12,13^ such as corpus callosum hypoplasia, to characteristic neurocognitive and behavioral symptoms^14–17^.

The FASD diagnosis is usually established by a group of professionals (physicians and psychologists) according to the clinical criteria^2,18,19^. The role of electroencephalography (EEG) as a part of the process of diagnosis is being debated. All three major diagnostic systems include seizure disorder^2,18,19^ as a proof of brain impairment, however they vary in interpretation whether it should be treated as “a hard neurological sign” or just a sign of distorted neurophysiology.

An increased incidence of both, epilepsy and abnormal findings in EEG was documented by several researchers^20–22^ described the characteristics of EEG patients with FASD. In^23^ the authors established an increased prevalence of both, epilepsy and seizure episodes among individuals with FASD in a retrospective database study. A prospective study presented by Boronat et. al. in^24^ demonstrated an increased incidence of EEG abnormalities even among patients without epilepsy, however, the study was performed without a control group.

All the aforementioned studies were limited to clinical qualitative analyses only. The quantitative electroencephalography (QEEG) is a type of EEG analysis that involves the use of mathematical algorithms and has brought some new possibilities of EEG signal evaluation, among other things, an analysis of a specific frequency band^25^. This technique enriches neurological interpretation, owing to the fact that it can show a more subtle dysfunction such as concentration and learning disorders or mental and emotional problems. The QEEG has its role in the neuropsychiatric disorders diagnosis, being: epilepsy, stroke, dementia, traumatic brain injury, depression, encephalopathy, learning and attention disorders and many more^26^.

To the best of our knowledge, no studies in which the quantitative analysis of EEG was applied in FASD have ever been published. Quantitative EEG has been studied in other neurodevelopmental disorders such as autism spectrum disorders (ASD)^27,28^ ADHD^27,29,30^ and other childhood conditions^31,32^. This study is aimed to investigate the characteristics of the bioelectric activity of the brain in resting-state conditions with quantitative EEG analysis methods.

## Methods

In order to test whether the signal features were statistically different between the control group and the FASD group, the three following types of two-sample statistical tests were performed:

In order to test whether the signal features were statistically different between the control group and the FASD group, the three following types of two-sample statistical tests were performed:

1. Two-sided Wilcoxon rank sum test (equivalent to the Mann-Whitney *U*-test; abbreviated as *MW* in the rest of the publication), which is a non-parametric test and in which the null hypothesis states that the medians of the two groups are equal;
2. Two-sample Kolmogorov-Smirnov test (abbreviated as *KS* in the rest of the publication), which is a non-parametric test, and in which the null-hypothesis states that the data from two groups come from the same distribution;
3. Kruskal–Wallis test (abbreviated as *KW* in the rest of the publication), which is a non-parametric test, and in which the null-hypothesis states that the data from two groups have equal distributions.

### Participants

The study was approved by the Ethical Comitte of Local Board of Physician in Cracow, Poland, on 16th July 2021, approval number: 12/*KBL*/*OIL*/2021. The experiments were conducted in accordance with the Helsinki Declaration. Appropriate consent form signed by a participant’s legal guardian or parent prior the experiments. We performed a pilot prospective study of patients newly diagnosed with FASD according to^2^ guidelines form. The patients were recruited in the FASD Diagnostic Center of St Louis Children Hospital in Cracow (Poland). The inclusion criteria were: FASD diagnosis according to the^2^ guidelines; age 6–13 years. The exclusion criteria were: a diagnosis of epilepsy, anti-epileptic treatment for other reasons. The group consisted of 12 children (females = 5, males = 7, mean age (*SD*) = 9.4 (1.8) years).

The controlled group consisted of children from the Department of Pediatric Gastroenterology (females = 8, males = 4, mean age (*SD*) = 9.2 (2.0) years), who were hospitalized for diagnostic purposes and had a diagnosis of a functional abdominal pain. The exclusion criteria were confirmed or unknown prenatal alcohol exposure, a diagnosis of epilepsy, anti-epileptic treatment for other reasons, other neurodevelopmental conditions such as ASD (autism spectrum disorder), ADHD (attention deficit hyperactivity disorder), a known structural brain defect, systemic conditions that could temporarily affect the EEG signal (active infectious disease, fever, dehydration).

### EEG recordings and analyses

The EEG recordings were obtained by the registered EEG technician at the EEG Laboratory of St Louis Children Hospital in Cracow (Poland) from August 2021 to October 2021. Before performing EEG recording, all procedures were explained to both a subject and a legal guardian or parent. The consent form signed by a legal guardian or parent was required prior to the EEG recording. Participants, especially from the experimental group, were asked to leave out the morning dose of any medication that could interfere with the EEG recording outcome. Participants were requested to be well-rested and relaxed on the test date. The recording lasted approximately 10 minutes and was carried out according to the same scheme: eyes open and eyes closed. All subjects were asked to lie still comfortably and in silence, remain relaxed, and instructed to stay awake with their eyes closed during the recording period. EEG was obtained using 19 tin electrodes, with an impedance level set below $5 \Omega$ were placed in an electrode cap based on the “10-20” system placed at typical anatomical landmarks. The signal was amplified with EEGDigiTrack Amplifier. Data were band-pass- ($0.3-70$ Hz) and notch (50/60 Hz) filtered, at a sampling rate 250 Hz. Data from the 19 channels were selected for the quantitative electroencephalographic analysis: **’Fp1’**, **’Fp2’**, **’F3’**, **’F4’**, **’F7’**, **’F8’**, **’C3’**, **’C4’**, **’T3’**, **’T4’**, **’P3’**, **’P4’**, **’T5’**, **’T6’**, **’O1’**, **’O2’**, **’Fz’**, **’Cz’** and **’Pz’**. Then, eyes-closed recording, artifact-free epochs were selected for analysis, using the QEEG module of DigiTrack Elmiko software. This QEEG module interprets the amplitude spectrum with peak-to-peak values in the range of absolute power in the frequency range of individual rhythms [$\mu V$], relative power that stands for the power of the individual rhythms is shown as a percentage of the sum of the power of all rhythms and rhythm comparisons presented as a ratio of the absolute strengths of the particular rhythms.

It is possible to differentiate the following EEG frequency ranges^33–36^:

- **Delta** ($$\delta $$): $$0-4$$ [Hz];
- **Theta** ($$\theta $$): $$4-8$$ [Hz];
- **Alpha** ($$\alpha $$): $$8-12$$ [Hz];
- **Beta** $$_{1}$$ ($$\beta _{1}$$): $$12-18$$ [Hz];
- **Beta** $$_{2}$$ ($$\beta _{2}$$): $$18-30$$ [Hz];
- **Gamma** ($$\gamma $$): $$30+$$ [Hz];

All analyses were performed with the Matlab 2021 software. Separately for each location and for each frequency range and for Delta/Beta and Theta/Beta power ratio was calculated as new variables by dividing the power of the slower frequency by the power of the faster frequency. We have calculated the correlation coefficient to explore the correlation between EEG absolute power in all locations and difference scores of hyperactivity and attention deficit for all frequency bands and power ratios (Delta/Beta and Theta/Beta).

To test whether the signal features were statistically different between the control group and the FASD group, as mentioned above—three types of two-sample statistical tests were performed: two-sided Wilcoxon rank sum test (MW), two-sample Kolmogorov-Smirnov test (KS), Kruskal–Wallis test (KW). If all three (MW, KS and KW) tests indicated a significant difference between groups, at 0.05 significance level, data were further analyzed. Full tables of *p*-values for MW, KS and KW tests were included in Appendix.

### Ethical approval and informed consent

Written informed consent was obtained from a parent or legal guardian prior to EEG recording and oral assent was obtained form children. Appropriate approval from the Ethical Comitte of Local Board of Physician in Cracow (Poland) was obtained on 16th July 2021 (12/*KBL*/*OIL*/2021). The study was carried out in accordance with the Helsinki Declaration.

## Results

**Figure 1:**
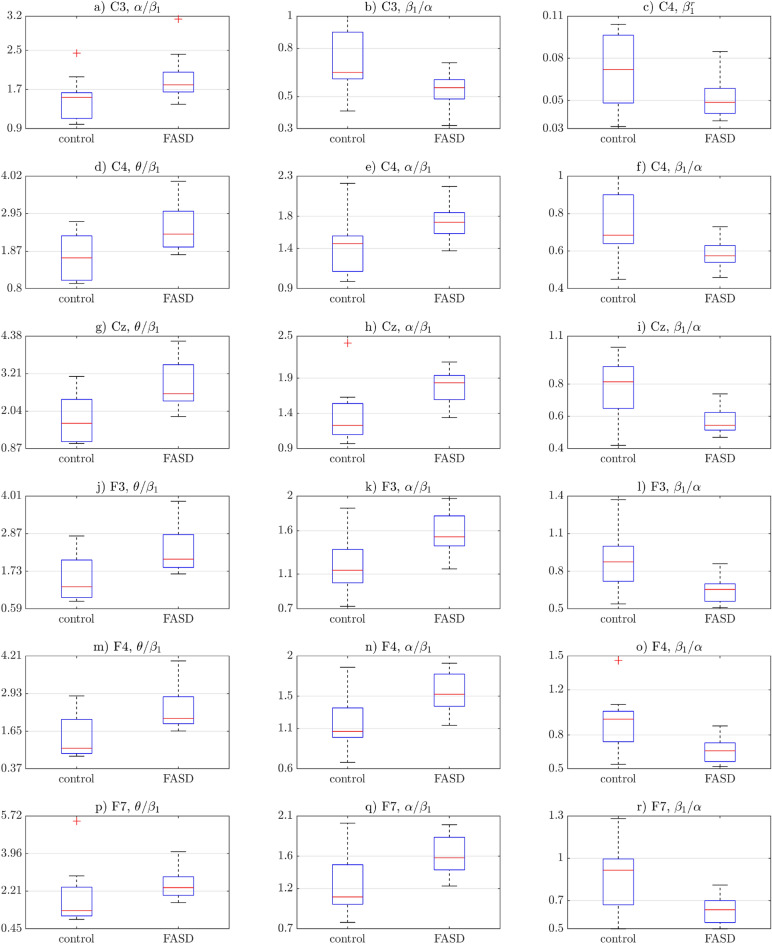
The first part of statistically significant features’ boxplots. Blue box indicates the interquartile range, while whiskers indicate the rest of the values not considered outliers. Outliers are marked with red crosses and red lines show the median. See the second part on Fig. 2.

**Figure 2:**
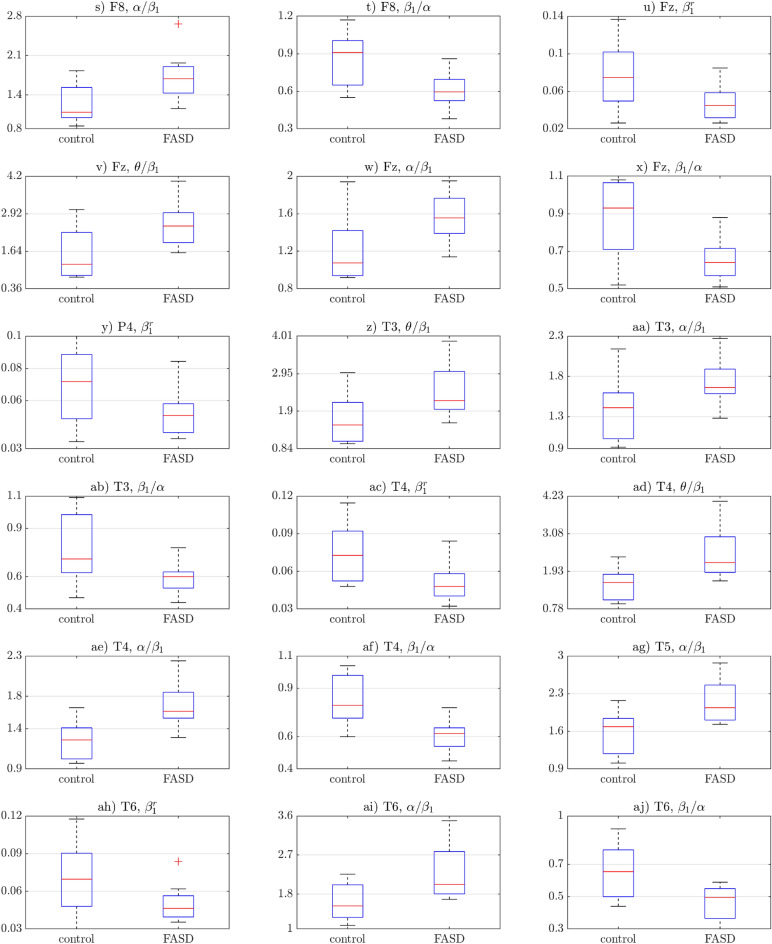
The second part of statistically significant features’ boxplots. See the first part on Fig. 1.

**Figure 3:**
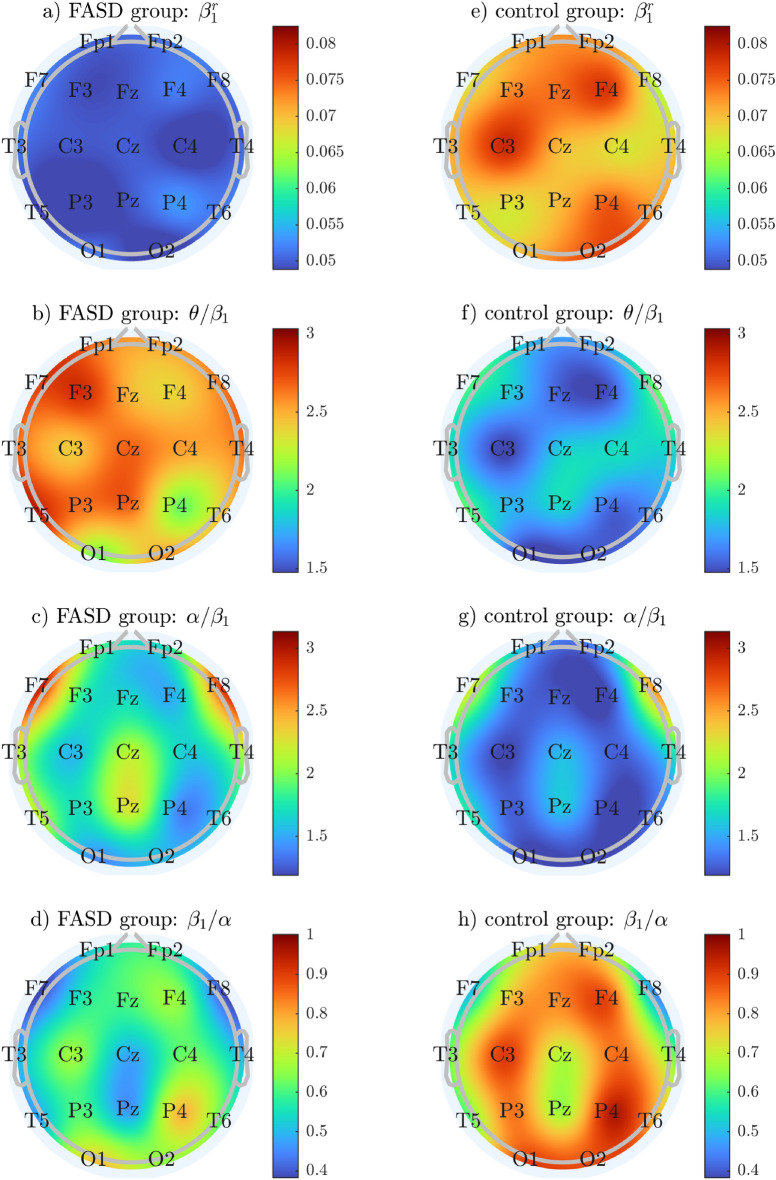
Topological plots of statistically significant features. Left and right column plots were obtained for FASD and control groups, respectively.

Features, which proved to be significantly different between groups, were compared. Differences between their mean values were shown in Table 1. Boxplots of significantly different groups were illustrated with Figs. 1 and 2. Topological plots of statistically significant features were included in Fig. 3. Note, that color-maps in particular rows (i.e., for particular features) were normalized to the same range, allowing easier comparison between groups.

**Table 1:** Differences between the mean values of the control group and the mean values of the FASD group.

el.	$\beta _1^r$	$\theta$/$\beta _1$	$\alpha$/$\beta _1$	$\beta _1$/$\alpha$
C3	ns	ns	$-$ 0.433	0.178
C4	0.021	-0.834	-0.325	0.164
Cz	ns	$-$ 1.028	$-$ 0.431	0.219
F3	ns	$-$ 0.883	$-$ 0.358	0.224
F4	ns	$-$ 0.963	$-$ 0.376	0.243
F7	ns	$-$ 0.643	$-$ 0.355	0.222
F8	ns	ns	$-$ 0.461	0.238
Fz	0.030	$-$ 1.045	$-$ 0.365	0.233
P4	0.019	ns	ns	ns
T3	ns	$-$ 0.810	$-$ 0.352	0.188
T4	0.025	$-$ 0.949	$-$ 0.439	0.223
T5	ns	ns	$-$ 0.598	ns
T6	0.021	ns	$-$ 0.661	0.194

Note, that only statistically significant differences were included; *ns* stands for not-significant.

Figure 1a shows that for the **C3** electrode, the $\alpha /\beta _{1}$ ratio was greater than 1 for the majority of values in both groups, meaning that regardless of the group, the $\alpha$ waves are more prominent than the $\beta _{1}$ waves. However, in the FASD group, this phenomenon proved to be greater. This result has been further confirmed by the $\beta _{1}/\alpha$ ratio in the same electrode (**C3**) (see Fig. 1b).

In the study group the above mentioned ratio proved to be lower than for the control group. Also, the majority of values are lower than 1, meaning that for both groups, again, $\alpha$ waves have greater absolute amplitude than the $\beta _{1}$ waves. Similar results can be observed in the opposite electrode—**C4** (Fig. 1e,f) and in the **Cz** electrode (Fig. 1h,i).

Results similar to those mentioned above were also observed in frontal electrodes: **F3** (Fig. 1k,l), **F4** (Fig. 1n,o), **Fz** (Fig. 2w,x), **F7** (Fig. 1q,r) and **F8** (Fig. 2s,t). Note, however, that for those electrodes, values between the FASD participants and the control group have not been as consistent, i.e. more values of the $\alpha /\beta _{1}$ ratio were lower than 1, particularly in the control group. Accordingly, the inverse ratio (i.e. $\beta _{1}/\alpha$) more often than in the central electrodes was greater than 1. It means that for the frontal electrodes in the control group, the $\beta _{1}$ waves proved to be more prominent than the $\alpha$ waves in some cases.

In the temporal electrodes, i.e. **T3** (Fig. 2aa,ab), **T4** (Fig. 2ae,af), **T5** (Fig. 2ag) and **T6** (Fig. 2ai,aj), the values of the $\alpha /\beta _{1}$ ratio were more similar to those obtined from the central electrodes. Note, that for the **F5** electrode, the $\beta _{1}/\alpha$ ratio differences between groups were not statistically significant (null hypothesis has not been rejected by the Kolmogorov-Smirnov test, see Table 2 in the Appendix).

The relative values of the $\beta _{1}$ waves proved to be statistically different between the groups in the **Fz** electrode (Fig. 2u) and some right-hemisphere electrodes, i.e. **C4** (Fig. 1c), **P4** (Fig. 2y), **T4** (Fig. 2ac) and **T6** (Fig. 2ah). In all cases, patients with the FASD obtained lower values.

The $\theta /\beta _{1}$ ratio revealed statistically significant differences in electrodes **C4** (Fig. 1d), **Cz** (Fig. 1g), **F3** (Fig. 1j), **F4** (Fig. 1m), **F7** (Fig. 1p), **Fz** (Fig. 2v), **T3** (Fig. 2z) and **T4** (Fig. 2ad). It is worth noting that for the mentioned electrodes, every FASD patient had the $\theta /\beta _{1}$ ratio greater than 1. It proves, that the $\theta$ waves were much more prominent than the $\beta _{1}$ waves. It can also be observed in the control group, though not as commonly. Additionally, the $\theta /\beta _{1}$ ratios proved to be generally greater in the FASD group.

## Discussion

In the current study, we established the dominance of the alpha rhythm over the beta rhythm in people with FASD compared to those from the control group, mostly in frontal and temporal locations. As it has been previously shown—an increase in the alpha power is associated with a decrease in brain tissue metabolism^37–42^. This finding is consistent with current knowledge on the pathological processes resulting from the PAE, as the PAE affects fetal brain in various ways^43^ and the influence on the neurometabolic is one of the effects previously described e.g. in:^44^.

In particular, the influence of the PAE on the glucose uptake by neurons was described in^45,46^. Furthermore, it was established that the patients with FASD have a decreased brain volume^47^ compared to those from the control group, which may also contribute to differences in brain tissue metabolism reflected as increased alpha power. Impaired brain growth was predominantly observed in frontal lobes^48,49^ which is consistent with our observations carried out during this study.

Meiers et al. established that the distribution of alpha rhythm at rest, called alpha asymmetry, is related to neglect and alcohol abuse in adolescents^50^. Children with FASD are often the subjects of abuse and neglect^51^. As our data present a mean activity in each channel, this effect could also contribute to the differences in alpha rhythm between the FASD group and the healthy controls. However, the relationship between alterations in QEEG signals and neglect has been presented in two studies only (see:^50^ and^52^) and little is known about the stability of this phenomenon. Nooner et al.^52^ established that the characteristic pattern was observed in QEEG among patients who had been recently subjected to neglect. It is impossible to exclude that patients from our FASD group have a history of abuse or neglect; however, they presented in a setting with adoptive or foster parents and their legal and psychosocial situation had been stable for a significant amount of time, so recent exposure to adverse life events can be excluded.

Moreover, an increased theta/beta ratio among patients with FASD seems to be an important finding, as it has been observed that a higher baseline theta/beta ratio occurs in individuals with ADHD^53,54^. Researchers are still debating whether this parameter can be treated as a biomarker of ADHD^55^. As the ADHD is treated as an important comorbidity of individuals with FASD^56–58^. However, the ADHD and FASD partially share the clinical picture, especially in terms of attention deficit^59^ and a misdiagnosis of a non-dysmorphic FASD being treated as ADHD is not uncommon^60,61^.

We assume that the deficits observed in both, conditions contribute to similar findings in EEG; however, using this parameter in clinical practice would make the differential diagnosis even more challenging. As neuropsychological problems in FASD differ between patients (see:^59^) with FASD and attention deficit (with or without a diagnosis of ADHD) could have contributed to the finding we present. More studies that could correlate clinical data, especially the results of neuropsychological evaluation, should be performed to confirm this effect. Interestingly, theta/beta ratio was also linked to the executive functions. It has been established that a lower theta/beta index is characteristic for developing children with higher levels of executive functions^62^. The lower theta/beta ratio in the front-line was associated with the greater inhibitory control among adults^63,64^.

Generally, an increased theta/beta ratio is believed to exhibit a lower tendency to self-reported attention control^65,66^, therefore our finding is fully consistent with the knowledge regarding the neuropsychological issues characteristic for FASD patients^67^. This is also because the cognitive deficits, in particular those related with executive functions, are the most important part of the FASD clinical picture^68,69^ and significantly contribute to the patients’ disability^70^.

The exact mechanism by which alcohol affects the fetal nervous system remains unknown^43^. However, most of the mechanisms by which PAE affects the central nervous system remain and can potentially contribute to its abnormal bioelectric activity. Firstly, the neurotoxic effect of alcohol depends on cell damage, since alcohol promotes both necrosis and apoptosis of neurons^44^. In addition to this effect, alcohol affects cell connectivity, notably reducing the density of glutamate receptors (NMDA) and inhibiting the growth of serotonergic neurons^44,71,72^. The influence of PAE on cellular metabolism that reduces glucose uptake is another mechanism that can contribute to impaired brain function that could be reflected in abnormal bioelectric activity.

Moreover, it has been established that the PAE is associated with selected structural brain defects such as corpus callosum hypoplasia^13^ and the evidence supports the crucial role of the corpus callosum in the propagation of bioelectrical impulses^73^. The patients who participated in the study did not have brain imaging performed, so the presence of structural brain defects can not be confirmed; however, even the defects in the corpus callosum microstructure, not identifiable by brain imaging, can be responsible for the observed effect. Furthermore, as reported by Dylag et al. in^74^, delayed myelinization of cortical neurons can be observed among individuals with FASD, what might reflect the slow maturation of the neurons in this group of patients^75^. As the velocity of impulse propagation depends on the myelinization^76^ this phenomenon can also justify our findings.

This is one of the first, if not the first study toward exploring the role of QEEG in FASD diagnosis, as this research establishes a quantitative framework to detect QEEG findings, which may be characteristic for the FASD^21^. The study was limited by the absence of clinical data, especially the history of possible neglect or abuse and the results of neuropsychological tests. Furthermore, the lack of data on comorbidities such as ADHD that could complicate the clinical picture and can contribute to alterations in EEG signals can be considered a study limitation.Furthermore, extending the analysis of the neuropsychological data could lead to a more broad spectrum of conclusions and is considered to be carried out in the future.

It should be also mentioned that the physiological variability of the EEG signals among children may affect the final results of the QEEG analysis^27^. The prospective recruitment of the patients and the controlled setting of the experiment used in our pilot study was applied to minimize the risk of potential bias. Caution must be taken in generalizing our results because of the relatively small sample size included in our study. However, this is a pilot study, which provided the promising results, the research will continue. Notwithstanding the relatively limited sample, this work offers valuable insights into the characteristics of bioelectrical activity of the brain in individuals with FASD with quantitative EEG analysis. Further research is required to establish the diagnostic roles of QEEG tools in FASD diagnosis.

## Conclusions

The purpose of the current research was to determine the characteristics of the QEEG findings among individuals with FASD. The research has also shown that an increased theta/beta ratio especially in frontal parts of the brain characterizes FASD patients in comparison to the control group. The second major finding was that alpha waves dominate over beta waves, especially in the frontal and temporal parts of the brain. The results of this research support the idea that QEEG might be a cheap and useful diagnostic tool for establishing the diagnosis of FASD. There is abundant room for further progress toward determining the role of quantitative EEG analysis in the FASD diagnosis, especially in terms of other frequency bands analysis, coherence, analysis of connectivity and spatial correlation.

## Supplementary Information

Supplementary Information.

## Data Availability

Data available upon written request from the corresponding author.
