# Supplementary material for: Initial study on quantitative electroencephalographic analysis of bioelectrical activity of the brain of children with fetal alcohol spectrum disorders (FASD) without epilepsy
Source: Sci Rep. 2023 Jan 3;13:109. doi: 10.1038/s41598-022-26590-4 (PMC9810692; doi:10.1038/s41598-022-26590-4)
Supplement: Supplementary file 1 — Supplementary Information. [file 41598_2022_26590_MOESM1_ESM.pdf]

Appendix – Additional Tables

”Initial Study on Quantitative Electroencephalographic Analysis of Bioelectrical Activity of the Brain of Children with Fetal Alcohol Spectrum Disorders (FASD) without Epilepsy”

November 26, 2022

Supplementary Table 1: p-values of the Wilcoxon Rank-Sum test (Mann-Whitney U-test). The null-hypothesis states that the samples come from the distributions with equal medians. Values less than 0.05 are in bold.

el.	δ^a	δ^r	θ^a	θ^r	α^a	α^r	β_1^a	β_1^r	β_2^a	β_2^r	δ/θ	θ/α	β_1/β_2	θ/β_1	α/θ	α/β_1	β_1/α
C3	0.34	0.19	0.37	0.13	0.71	0.98	0.54	0.07	0.75	0.54	0.44	0.26	0.75	**0.02**	0.24	**0.01**	**0.01**
C4	0.47	0.18	0.37	0.16	1.00	0.39	0.51	**0.04**	1.00	0.54	0.37	**0.04**	0.86	**0.01**	**0.04**	**0.01**	**0.01**
Cz	0.26	0.14	0.21	0.06	0.80	0.44	0.47	**0.03**	0.89	0.47	0.30	**0.04**	0.75	**0.01**	**0.04**	**0.00**	**0.00**
F3	0.51	0.31	0.24	0.18	0.98	0.37	0.58	0.05	0.89	0.62	0.67	**0.04**	0.84	**0.01**	0.05	**0.01**	**0.01**
F4	0.29	0.21	0.16	0.16	0.89	0.37	0.51	0.05	0.58	0.44	0.34	**0.02**	0.73	**0.01**	**0.02**	**0.01**	**0.01**
F7	0.47	0.40	0.31	0.31	0.98	0.62	0.47	0.07	0.62	0.51	0.91	0.16	0.80	**0.04**	0.17	**0.02**	**0.02**
F8	0.44	0.54	0.19	0.14	0.62	0.75	0.47	0.13	0.89	0.62	1.00	0.27	0.69	0.06	0.26	**0.01**	**0.01**
Fp1	0.71	0.80	0.51	0.40	0.93	0.62	0.67	0.14	0.98	0.89	0.71	0.17	0.91	0.10	0.17	0.09	0.10
Fp2	0.58	0.58	0.40	0.44	0.80	0.26	0.89	0.05	0.93	0.75	0.98	0.09	0.82	0.08	0.08	**0.04**	**0.04**
Fz	0.19	0.21	0.31	0.26	0.98	0.24	0.44	**0.02**	0.89	0.62	0.21	**0.01**	0.50	**0.01**	**0.01**	**0.01**	**0.01**
O1	0.34	0.26	0.31	0.37	0.11	0.62	0.58	0.09	1.00	0.29	0.51	0.82	0.84	0.05	0.75	**0.04**	**0.04**
O2	0.62	0.44	0.84	0.58	0.31	0.54	0.47	**0.04**	0.98	0.71	0.40	0.73	0.75	0.08	0.77	0.31	0.33
P3	0.34	0.21	0.67	0.42	0.54	0.98	0.44	0.05	0.93	0.54	0.44	0.44	0.82	**0.03**	0.44	0.08	0.07
P4	0.44	0.34	0.54	0.21	0.84	0.80	0.51	0.05	0.89	0.58	0.39	0.40	0.73	**0.02**	0.42	0.11	0.12
Pz	0.26	0.26	0.62	0.37	0.80	0.89	0.44	**0.04**	0.84	0.54	0.37	0.39	0.84	**0.03**	0.39	0.08	0.08
T3	0.75	0.51	0.51	0.26	1.00	0.31	0.37	0.05	0.80	0.58	0.89	0.11	0.47	**0.02**	0.10	**0.01**	**0.01**
T4	0.37	0.37	0.31	0.31	0.89	0.13	0.29	**0.00**	1.00	0.51	0.34	0.10	0.52	**0.00**	0.09	**0.00**	**0.00**
T5	0.47	0.21	0.47	0.19	0.34	0.77	0.47	**0.04**	0.62	0.47	0.49	0.64	0.93	**0.01**	0.64	**0.00**	**0.00**
T6	0.37	0.24	0.54	0.18	0.26	0.67	0.44	**0.03**	0.84	0.47	0.31	0.89	0.93	**0.01**	0.89	**0.01**	**0.01**

Supplementary Table 2: p-values of the Kolmogorov-Smirnov test. The null-hypothesis states that the samples come from the same continuous distribution. Values less than 0.05 are in bold.

el.	δ^a	δ^r	θ^a	θ^r	α^a	α^r	β_1^a	β_1^r	β_2^a	β_2^r	δ/θ	θ/α	β_1/β_2	θ/β_1	α/θ	α/β_1	β_1/α
C3	0.43	0.43	0.19	0.07	0.79	0.99	0.43	0.19	0.79	0.43	0.79	0.43	0.79	0.07	0.43	**0.02**	**0.02**
C4	0.43	0.19	0.07	0.07	0.79	0.79	0.43	**0.02**	0.99	0.43	0.79	0.19	0.79	**0.02**	0.43	**0.02**	**0.02**
Cz	0.43	0.43	0.07	0.07	0.43	0.43	0.43	0.07	0.99	0.43	0.79	0.19	0.79	**0.02**	0.19	**0.01**	**0.01**
F3	0.43	0.43	**0.02**	0.07	0.43	0.43	0.43	0.07	0.99	0.43	0.79	0.19	0.79	**0.01**	0.19	**0.01**	**0.01**
F4	0.43	0.43	0.07	0.07	0.43	0.43	0.43	0.07	0.43	0.43	0.43	0.07	0.79	**0.02**	0.07	**0.02**	**0.02**
F7	0.43	0.43	0.19	0.19	0.43	0.43	0.43	0.07	0.79	0.43	0.79	0.43	0.79	**0.02**	0.43	**0.02**	**0.02**
F8	0.43	0.79	0.07	0.07	0.43	0.79	0.79	0.07	0.99	0.43	0.43	0.19	0.79	**0.02**	0.19	**0.02**	**0.02**
Fp1	0.43	0.79	0.43	0.19	0.79	0.99	0.43	0.07	0.99	0.43	0.43	0.19	0.79	0.07	0.19	0.07	0.07
Fp2	0.43	0.79	0.19	0.19	0.43	0.43	0.79	0.19	0.99	0.43	0.79	0.19	0.79	0.19	0.19	0.07	0.07
Fz	0.43	0.79	0.19	0.19	0.43	0.43	0.79	**0.02**	0.99	0.43	0.19	0.07	0.79	**0.01**	0.07	**0.01**	**0.01**
O1	0.43	0.43	0.43	0.43	0.43	0.43	0.43	0.19	0.99	0.19	0.79	0.79	0.99	0.43	0.79	0.07	0.07
O2	0.43	0.43	0.43	0.43	0.43	0.79	0.43	0.07	0.99	0.43	0.79	0.99	0.79	0.19	0.99	0.43	0.43
P3	0.43	0.43	0.43	0.43	0.79	0.79	0.43	0.07	0.99	0.43	0.79	0.43	0.79	0.19	0.43	0.19	0.19
P4	0.43	0.19	0.43	0.19	0.79	0.79	0.43	**0.02**	0.99	0.43	0.79	0.79	0.79	0.19	0.79	0.19	0.19
Pz	0.43	0.43	0.43	0.19	0.79	0.99	0.43	0.19	0.99	0.43	0.79	0.43	0.79	0.19	0.43	0.07	0.19
T3	0.43	0.79	0.43	0.07	0.43	0.19	0.19	0.07	0.79	0.43	0.99	0.43	0.43	**0.02**	0.43	**0.02**	**0.02**
T4	0.43	0.19	0.07	0.07	0.43	0.07	0.43	**0.02**	0.99	0.19	0.43	0.43	0.43	**0.01**	0.43	**0.01**	**0.01**
T5	0.43	0.19	0.43	0.19	0.79	0.79	0.43	0.07	0.43	0.19	0.79	0.79	0.79	0.07	0.79	**0.02**	0.07
T6	0.43	0.19	0.43	0.19	0.43	0.43	0.43	**0.02**	0.99	0.43	0.79	0.99	0.79	0.07	0.99	**0.02**	**0.02**

Supplementary Table 3: p-values of the Kruskal–Wallis test. The null-hypothesis states that the samples come from the same distribution. Values less than 0.05 are in bold.

el.	δ^a	δ^r	θ^a	θ^r	α^a	α^r	β_1^a	β_1^r	β_2^a	β_2^r	δ/θ	θ/α	β_1/β_2	θ/β_1	α/θ	α/β_1	β_1/α
C3	0.33	0.18	0.36	0.12	0.69	0.95	0.53	0.07	0.73	0.53	0.42	0.25	0.73	**0.02**	0.23	**0.01**	**0.01**
C4	0.45	0.17	0.36	0.15	1.00	0.37	0.49	**0.03**	1.00	0.53	0.36	**0.04**	0.84	**0.01**	**0.04**	**0.01**	**0.01**
Cz	0.25	0.13	0.20	0.06	0.77	0.42	0.45	**0.02**	0.86	0.45	0.29	**0.04**	0.73	**0.01**	**0.03**	**0.00**	**0.00**
F3	0.49	0.30	0.23	0.17	0.95	0.36	0.56	**0.04**	0.86	0.60	0.64	**0.04**	0.82	**0.01**	**0.04**	**0.01**	**0.01**
F4	0.27	0.20	0.15	0.15	0.86	0.36	0.49	**0.04**	0.56	0.42	0.33	**0.02**	0.71	**0.01**	**0.02**	**0.01**	**0.01**
F7	0.45	0.39	0.30	0.30	0.95	0.60	0.45	0.07	0.60	0.49	0.89	0.15	0.77	**0.04**	0.16	**0.02**	**0.01**
F8	0.42	0.53	0.18	0.13	0.60	0.73	0.45	0.12	0.86	0.60	1.00	0.26	0.66	0.05	0.25	**0.01**	**0.01**
Fp1	0.69	0.77	0.49	0.39	0.91	0.60	0.64	0.13	0.95	0.86	0.69	0.16	0.89	0.09	0.16	0.09	0.09
Fp2	0.56	0.56	0.39	0.42	0.77	0.25	0.86	0.05	0.91	0.73	0.95	0.08	0.80	0.07	0.08	**0.04**	**0.04**
Fz	0.18	0.20	0.30	0.25	0.95	0.23	0.42	**0.02**	0.86	0.60	0.20	**0.01**	0.49	**0.01**	**0.01**	**0.01**	**0.01**
O1	0.33	0.25	0.30	0.36	0.11	0.60	0.56	0.08	1.00	0.27	0.49	0.80	0.82	0.05	0.73	**0.04**	**0.04**
O2	0.60	0.42	0.82	0.56	0.30	0.53	0.45	**0.04**	0.95	0.69	0.39	0.71	0.73	0.08	0.75	0.30	0.31
P3	0.33	0.20	0.64	0.40	0.53	0.95	0.42	0.05	0.91	0.53	0.42	0.42	0.80	**0.02**	0.42	0.07	0.07
P4	0.42	0.33	0.53	0.20	0.82	0.77	0.49	**0.04**	0.86	0.56	0.37	0.39	0.71	**0.02**	0.40	0.11	0.11
Pz	0.25	0.25	0.60	0.36	0.77	0.86	0.42	**0.03**	0.82	0.53	0.36	0.37	0.82	**0.03**	0.37	0.07	0.07
T3	0.73	0.49	0.49	0.25	1.00	0.30	0.36	0.05	0.77	0.56	0.86	0.11	0.45	**0.02**	0.09	**0.01**	**0.01**
T4	0.36	0.36	0.30	0.30	0.86	0.12	0.27	**0.00**	1.00	0.49	0.33	0.09	0.51	**0.00**	0.09	**0.00**	**0.00**
T5	0.45	0.20	0.45	0.18	0.33	0.75	0.45	**0.04**	0.60	0.45	0.47	0.62	0.91	**0.01**	0.62	**0.00**	**0.00**
T6	0.36	0.23	0.53	0.18	0.25	0.64	0.42	**0.03**	0.82	0.45	0.30	0.86	0.91	**0.01**	0.86	**0.01**	**0.01**
